# Antimicrobial Activity of Al_2_O_3_, CuO, Fe_3_O_4_, and ZnO Nanoparticles in Scope of Their Further Application in Cement-Based Building Materials

**DOI:** 10.3390/nano8040212

**Published:** 2018-03-31

**Authors:** Pawel Sikora, Adrian Augustyniak, Krzysztof Cendrowski, Paweł Nawrotek, Ewa Mijowska

**Affiliations:** 1 Faculty of Civil Engineering and Architecture, West Pomeranian University of Technology, Szczecin, Al. Piastow 50, 71-310 Szczecin, Poland; pawel.sikora@zut.edu.pl; 2 Department of Immunology, Microbiology and Physiological Chemistry, Faculty of Biotechnology and Animal Husbandry, West Pomeranian University of Technology, Szczecin, Al. Piastów 45, 70-311 Szczecin, Poland; pawel.nawrotek@zut.edu.pl; 3 Nanomaterials Physicochemistry Department, Faculty of Technology and Chemical Engineering, West Pomeranian University of Technology, Szczecin, Al. Piastow 45, 70-311 Szczecin, Poland; kcendrowski@zut.edu.pl (K.C.); ewa.mijowska@zut.edu.pl (E.M.)

**Keywords:** nanomaterials evaluation, microbial models, toxicity, metal oxides, cement-based composites

## Abstract

Nanoparticles were proposed as antibacterial cement admixtures for the production of cement-based composites. Nevertheless, the standards for evaluation of such admixtures still do not indicate which model organisms to use, particularly in regard to the further application of material. Apart from the known toxicity of nanomaterials, in the case of cement-based composites there are limitations associated with the mixing and dispersion of nanomaterials. Therefore, four nanooxides (Al_2_O_3_, CuO, Fe_3_O_4_, and ZnO) and seven microorganisms were tested to initially evaluate the applicability of nanooxides in relation to their further use in cement-based composites. Studies of nanoparticles included chemical analysis, microbial growth kinetics, 4- and 24 h toxicity, and biofilm formation assay. Nanooxides showed toxicity against microorganisms in the used concentration, although the populations were able to re-grow. Furthermore, the effect of action was variable even between strains from the same genus. The effect of nanoparticles on biofilms depended on the used strain. Gathered results show several problems that can occur while studying nanoparticles for specific further application. Proper protocols for nanomaterial dispersion prior the preparation of cement-based composites, as well as a standardized approach for their testing, are the fundamental issues that have to be resolved to produce efficient composites.

## 1. Introduction

In recent years, nanotechnology has gained much attention, mostly due to the versatile applications of its products in industry. Nanoparticles, including metals and oxides, found application in electronics, cosmetics, food industry, agriculture, and building materials (especially cement-based composites) [1]. The production of cementitious composites (cement mortars and concretes) is one of the most important branches in building materials. In 2012, Imbabi et al. [2] showed that the world market of ordinary Portland Cement reached 3.6 billion metric tons annually. According to the provided estimation, the volume will reach around 5 bln metric tons by 2030. Such demand on the material leads to the developmental works on novel materials, admixtures, plasticizers, etc. [2,3]. 

Mixing even a small amount of nanomaterials with cement-based constituents (cement, water, and aggregate) can considerably affect the mechanical properties and durability of cement-based composites, as well as provide additional functional properties such as self-sensing, self-healing, or electrical resistivity [4]. Moreover, particular research efforts were focused on the development of self-cleaning composites, e.g., containing TiO_2_ nanoparticles that could be effectively used as photocatalysts [5,6,7]. The utility of cement-based materials as the photocatalyst-supporting media is feasible and very effective because of its strong binding. In addition, the porous structure of cement matrix facilitates the reaction between photocatalyst and pollutants [8]. In spite of the fact that main focus is set on photocatalytically active surfaces (including cement mortars containing TiO_2_ nanoparticles), various other photocatalytic (e.g., ZnO, CuO,) and non-photocatalytic nanomaterials (e.g., Al_2_O_3_, Fe_3_O_4_, AgO) exhibiting antimicrobial activity were also studied [7,9,10,11]. The incorporation of such nanomaterials can contribute to the development of cement-based composites that would be applicable in places where UV/solar light is limited or unavailable, such as sewer systems, waste water tanks, etc. However useful the nanoparticles are, authors sill argue about their impact on terrestrial and aquatic organisms, publishing studies based on diverse methods [3,12,13,14]. Despite the fact that there is much data on their potential to be antimicrobial agents [2,3,4,5], there are still no general guidelines about how to assess these nanoparticles before they are used in the industrial production of cementitious composites. Current regulations in the European Union are being expanded each year, although most of them regard food production, biocides, cosmetics, and medical applications [15]. Guidelines considering the evaluation of nanomaterials are still limited in spite of the procedures and standards that were established over the years by, e.g., International Organization for Standardization (ISO) and Japanese Industrial Standards (JIS). They were dedicated mostly to the evaluation of self-cleaning properties of semiconducting photocatalytic materials. These standards were successfully incorporated in numerous analyses on the self-cleaning capacity of cement-based composites [6,7,9,10,16]. 

Several microbial models (e.g., *Staphylococcus aureus*, *Escherichia coli*, and *Klebsiella pneumoniae*) were incorporated for studies on the antimicrobial performance of ceramic semiconducting photocatalytic or other materials that were manufactured through coating or mixing with photocatalysts [16]. Nevertheless, International standards can be applied specifically to the assessment of antibacterial activity on the photocatalytic ceramic materials and cannot be effectively used for the materials which are permeable or contain rough surfaces. Hence, various procedures are being developed to analyze the antimicrobial and antifungal properties of photocatalytic cementitious composites [3,5,8,17]. Nonetheless, no standardized procedures were established where non-photocatalytic cement-based surfaces were applied for the antimicrobial performance. This creates many discrepancies between studies in which authors use different techniques and equipment. There is a necessity to develop standardized methods for the evaluation of nanomaterials regarding their potential ecotoxicity. On that basis, diverse methods dedicated to different materials (e.g., polymer materials) are being developed or adapted [18,19,20,21].

Generally, microorganisms are a suitable model for studies considering nanomaterials in various aspects ranging from industrial applications to building materials, environmental protection, and agriculture [22]. Microbiological models can be useful in testing numerous nanomaterials from nanoparticles, through nanorods, to nanocomposites planned for the use in various applications [23,24,25,26]. In addition, there is no agreement in which bacterial strains are the most suitable for such evaluation. For example, Muynck et al. [21] evaluated the effect of antimicrobial cement-based surfaces on Gram-negative (G^−^) bacteria *Escherichia coli* and *Salmonella enterica,* and Gram positive (G^+^) bacteria *Listeria monocytogenes* and *Staphylococcus aureus,* while other authors limited their research to *E. coli* [3,8,17,27]. This makes available studies difficult to validate and analyze the test results. 

Despite the methodological problem associated with the analysis of cement-based composites bactericidal properties, there are also other issues that can impede the performance given by nanomaterials within the cementitious composites. Firstly, due to the dimension of concrete structures or area of mortars/plasters applied on the building surfaces, the amount of nanomaterial should be optimized in order to enhance its effectiveness, reduce the necessary amount of nanomaterial, and meet economic requirements [28]. The cost of additives should not significantly increase the value of cement-based composite [2]. Nanomaterials, such as SiO_2_, TiO_2_, Al_2_O_3_, Fe_3_O_4_, ZnO, and CuO, are favored, because they are relatively inexpensive, effortlessly manufactured, and broadly available. Commercially available nanomaterials are more preferred for practical application than ones synthesized in laboratory for technological reasons and because of the adaptive character of civil engineering [4]. Usually, in order to satisfy economic and technological requirements, the amount of nanomaterials incorporated into the cement-based composite should not exceed 5 wt % of cement mass. Therefore, methods to optimize the dosage of nanomaterials and fully exploit performance of nanomaterials in cementitious composites are still being sought [28,29,30]. 

Finally, the key issue in the incorporation of nanomaterials to cement-based composites is their proper dispersion in the cement matrix. Agglomeration of nanomaterials significantly decreases their chemical and physical activity, hindering their efficiency in cement matrix performance and antimicrobial activity [29,30]. Therefore, the proper dispersion of nanomaterials in the cement matrix is the key issue addressed by many researchers. Nanomaterials added in a bulk states do not provide sufficient dispersion; therefore, diverse methods are developed by researchers, including mechanical stirring, ultrasonication, and ball milling of nanoparticles [30]. Nevertheless, to disperse nanomaterial, a dispersion medium (most likely mixing water) is required. Because of the fact that mixing water in cement mortars and concretes forms the final properties, the water-to-cement ratio (w/c) practiced in civil engineering is lower or equal 0.5. This implies that a limited amount of water is available for dispersion. Moreover, the temperature of mixing water prior to its addition to dry components (cement and aggregates) must remain ambient; therefore, thermal processing of suspension should be avoided so that the cement hydration process is not interrupted [29,30,31,32]. 

**Figure 1:**
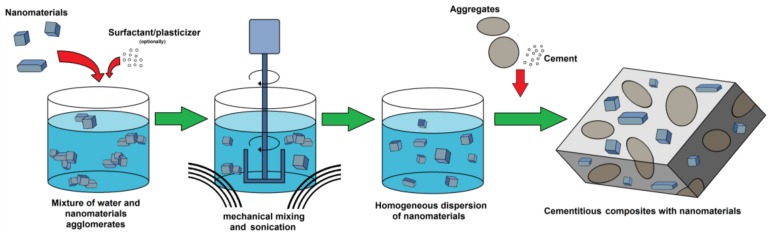
Schematic process of nanomaterials dispersing method commonly used in cement-based composite preparation.

Organic admixtures and different surfactant types [28] are incorporated to facilitate the dispersion process, thus achieving a certain dispersion state. Surface active agents are widely used to improve the homogeneity of dispersion because of the formation of aggregates around nanoparticles [33]. Such action is attributed to the containment of both hydrophilic and hydrophobic groups. The aggregation of surfactants around nanoparticles usually occurs in the form of micelles. The hydrophobic groups interact with the nanoparticles, whereas hydrophilic groups reduce the surface tension of water and thus increase the dispersion of nanomaterial. Unfortunately, many surfactants that are successfully used to disperse nanomaterials, e.g., in polymeric matrices, have been reported to affect the cement hydration kinetics, as well as negatively react with other admixtures. Therefore, methods for the incorporation of plasticizers and superplasticizers (especially polycarboxylate ether-based-PCE) that are compatible with cement have been widely evaluated as dispersants [29,30]. The typical nanomaterial dispersion process prior to the incorporation of cement-based composites is presented in Figure 1.

Nevertheless, at this developmental level, in the mass scale production of cement-based composites, the efficient sonication of high numbers of nanoparticles and subsequently facilitating the stable and satisfactory dispersion in cement-based composite is a considerable obstacle. Moreover, stabilization following the dissolution of agglomerates and maintaining the dispersed state seem to be very demanding. Thus, even with satisfactory dispersion, the re-agglomeration phenomena still can occur, leading to a significant change in the nanoparticle size distribution. This would likely decrease the performance of nanomaterials in cementitious composites [31,32,34]. Therefore, all of these elements should be also included in the phase of initial testing of proposed cement additives. 

Apart from the successful incorporation of non-photocatalytic nanomaterials into cementitious composites so far, there is missing data on methods for their application and evaluation, while existing papers do not agree about which microorganisms are the most suitable for such evaluation. Therefore, we aimed to contribute to the state of the art by evaluating the most popular commercially available metal oxide nanoparticles (Al_2_O_3_, CuO, Fe_3_O_4_, ZnO) used for the modification of cement-based composites on selected microbial models in a way that they would be likely used in industry. By conducting a series of tests, our goal was to present problems and observations associated with such studies in the scope of further use of nanoparticles in cement-based composites. 

## 2. Results

### 2.1. Evaluation of Nanoparticles

**Figure 2:**
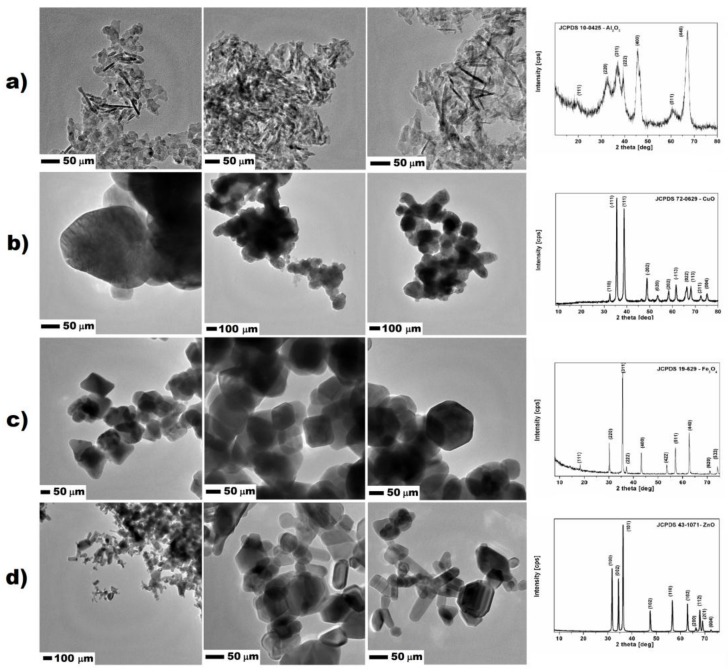
TEM microphotographs and XRD patterns of studied nanoparticles: (**a**) Al_2_O_3_; (**b**) CuO; (**c**) Fe_3_O_4_; and (**d**) ZnO.

Nanomaterials used for studies were purchased from Sigma Aldrich (Darmstadt, Germany). All nanomaterials were additionally characterized by the transmission electron microscopy (TEM) and X-ray diffraction (XRD). Aluminum oxide had regular shape and size, forming rod-, flake-shaped and formless nanoparticles. The average size of all nanomaterials was below 100 nm. XRD analysis confirmed that samples were composed only of aluminum oxide, which corresponded to the standard JCPDS 10-0425. The surface area of Al_2_O_3_ nanoparticles measured with the BET method was 110.6 m^2^/g. Aluminum oxide nanostructures had the highest surface area from all of the studied nanomaterials. Similarly, copper oxide expressed no uniformed shape and size. The nanoparticles were more spherical with size ranging from 100 to 250 nm. XRD analysis proved that the nanomaterial was composed only from the copper oxide, corresponding to the phase standard card JCPDS 72-0629. The surface area of CuO nanoparticles was lower and calculated to be 4.891 m^2^/g (measured with the BET method) because of the larger particles and the higher density of material. In the case of iron oxide nanoparticles, TEM images showed that they had uniformed size ranging from 50 to 150 nm, and cubic shape. According to the XRD analysis and data provided by the supplier, iron oxide nanoparticles were in the form of magnetite, which corresponded to standard card JCPDS 19-629. The surface area of iron oxide measured with the BET method was 27.08 m^2^/g. Zinc oxide nanoparticles characterization showed the composition of two uniformed shapes of nanoparticles nanorods and spherical nanostructures. The XRD analysis of zinc oxide structures corresponded to one standard card JCPDS 43-1071; therefore, the spherical nanoparticles were mostly in the amorphous form. The size of the nanostructures ranged from the 50 to 300 nm. MultiPoint BET method showed that the surface area of the zinc oxide was 14.11 m^2^/g. Except for the molecular mass and shape of the zinc oxide nanostructures, surface area-determining factors were important for their size ranging above 200 nm. The TEM images of studied nanomaterials together with XRD patterns are showed in Figure 2.

### 2.2. Growth Kinetics

**Figure 3:**
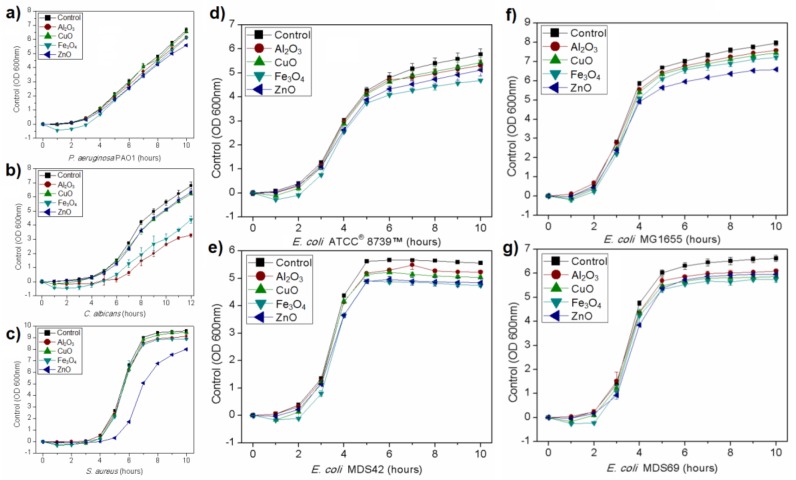
Growth kinetic curves of microorganisms treated with nanoparticles in comparison to control culture: (**a**) *P. aeruginosa*; (**b**) *S. aureus*; (**c**) *C. albicans*; (**d**–**g**) four different *E. coli* strains.

Growth kinetics curves were established for all microorganisms. All studied nanoparticles inhibited microbial growth in used concentration, although the result depended on the microorganism and nanomaterial. Results showing the growth kinetics curves of *P. aeruginosa*, *Staphylococcus aureus*, and *Candida albicans* are presented in Figure 3. The effect of studied nanoparticles was dependent on the strain that is shown in Figure 3 that presents the growth curves of four different strains of *Escherichia coli*. The used strains showed various responses to nanomaterials in the growth environment. The highest inhibitory effect on *E. coli* ATCC^®^ 8739™ had Fe_3_O_4_ nanoparticles while on *E. coli* MG1655 ZnO nanoparticles. The growth tendencies shown in Figure 3 were replicable. 

### 2.3. Acute Toxicity 4-h Test

**Figure 4:**
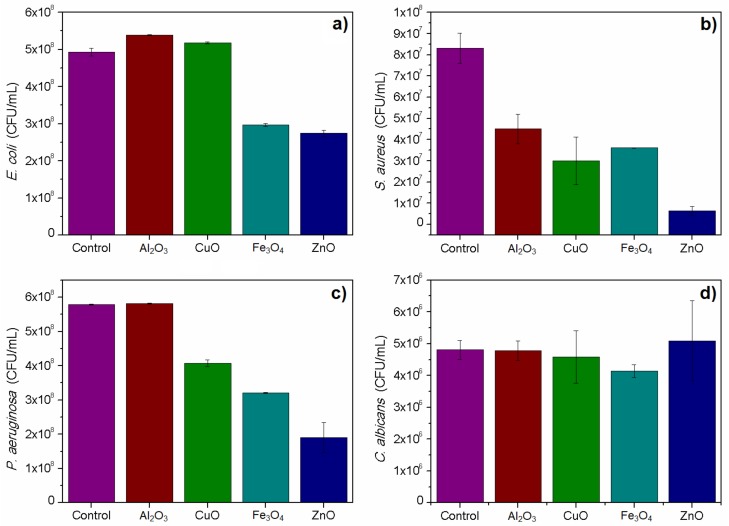
Plate count of cultures treated with nanomaterials in comparison to control samples: (**a**) *E. coli*; (**b**) *S. aureus*; (**c**) *P. aeruginosa* and (**d**) *C. albicans.*

The 4 h toxicity test confirmed the toxicity of studied nanoparticles on selected microbial models in selected dose. Relatively, the highest toxicity was obtained for ZnO nanoparticles. All used bacteria were susceptible to Fe_3_O_4_ and ZnO nanoparticles. Surprisingly, the test did not show toxicity of CuO nanoparticles on the used *E. coli* strain, which was confirmed in an additional round of experiments. *Candida albicans* viability was not significantly affected by studied nanomaterials, except for Fe_3_O_4_ nanoparticles, which caused a slight decrease in the viable cells count. The aluminum oxide nanoparticles were toxic in this test only against the used *S. aureus* strain. The results are presented in Figure 4. All described results were statistically significant with *p* < 0.05.

### 2.4. Toxicity in 24-h Test

**Figure 5:**
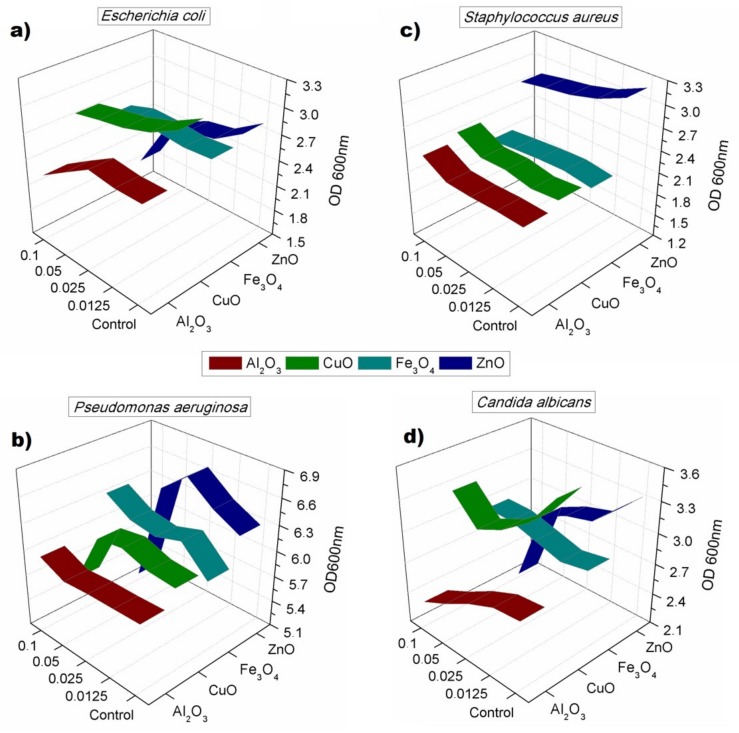
Optical density obtained for cultures after 24 h of incubation with four concentrations of nanoparticles: (**a**) *E. coli*; (**b**) *S. aureus*; (**c**) *P. aeruginosa*; (**d**) *C. albicans*.

The 24-h toxicity showed that the toxic effect of nanomaterials was not permanent, and most of cultures were able to re-grow after the 24-h incubation. Such phenomena occurred especially in case of *S. aureus,* which was able to re-grow after 24 h of incubation liquid medium, after showing susceptibility to ZnO nanoparticles in 4 h test. In general, the toxicity was noticed, especially in the case of ZnO nanoparticles, which resulted in lower OD values gained for all cultures with the highest activity against *Pseudomonas aeruginosa* and *Candida albicans*. *P. aeruginosa* showed signs of inhibition in the 24-h test caused by CuO nanoparticles. The concentration used for the toxicity test did not allow one to obtain minimal inhibitory concentration (MIC) for any of studied nanomaterials. Figure 5 shows described results on 3D plots that in each case show used nanoparticles, their concentration (beginning from 0 in case of the control samples), and the optical density gained after 24 h. Ribbons show the average OD measured after incubation time, which was compensated for in regard to initial culture (timepoint 0) and the noise given by nanomaterials. The experiments confirmed toxicity against *Candida albicans* observed on the growth kinetics curves. All described results were statistically significant with *p* < 0.05.

### 2.5. Biofilm Formation Assay

**Figure 6:**
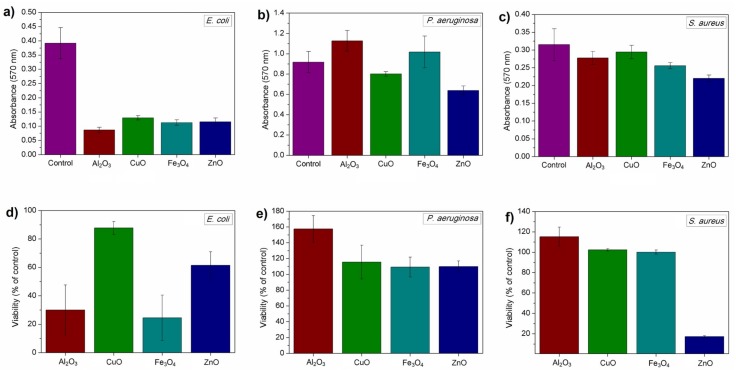
Biofilm biomass (upper row—(**a**–**c**)) and viability of cells in biofilms (bottom row—(**d**–**f**)) in relation to control sample.

Adherence was tested in the biofilm formation assay, which tested both the viability of cells forming biofilm and its biomass. Tested nanoparticles were able to reduce the formation of biofilms in studied bacteria, although there was no statistically significant difference between the samples of *C. albicans* (data not shown). Nanoparticles successfully affected the formation of bacterial biofilms. Similarly to previous experiments, results here were also different in terms of used bacterium. *E. coli* ATCC^®^ 8739™ biofilms were inhibited by all nanomaterials, although the viability of cells in biofilm was not completely reduced. Similarly to the results gained in 4-h toxicity test, CuO nanoparticles only slightly reduced the viability of cells. In that case, the biomass and the number of cells (viability) were lower than in the control samples. *P. aeruginosa* and *S. aureus* biofilms were significantly affected by ZnO nanoparticles, in which biomass was lower than in the controls; this occurred similarly with the viability of cells in the case of *S. aureus*. The viability of *P. aeruginosa* cells was comparable to the control sample with the exception of sample incubated with Al_2_O_3_ nanoparticles, in which it was significantly higher. These nanoparticles reduced the biofilm and viability of *E. coli*. The results are presented in Figure 6. All described results were statistically significant with *p* < 0.05.

## 3. Discussion

Nanomaterials are gaining significant interest in the field of modification of cementitious composites; nevertheless, the amount of nanomaterial should be optimized in order to enhance its effectiveness, reduce the necessary amount of nanomaterial, and meet the economical requirements to apply them in concrete structures or mortars/plasters applied on building surface. Unfortunately, the addition of fine particles into cementitious composite leads to their agglomeration. In current studies, sonicated suspensions of nanomaterials were used, which were additionally characterized apart from data given by the manufacturer (see Figure 2). Due to the increase of specific surface area with the decrement of particles diameter, van der Waals, electrostatic, and magnetic forces become more dominant compared to gravitational-shear forces, which lead to the agglomeration and formation of interconnected flocs [28,29,30]. Therefore, when nanoparticles would not be uniformly dispersed in the suspension (prior mixing with dry components), further dispersion even with the use of high shear cement mixers could be demanding. As stated by Korayem et al. [29] ‘*the ideal dispersion can be described as the state in which nanoparticles are completely separated from each other and no clusters or agglomerates exists’*. Nevertheless, obtaining the complete dispersion of nanomaterial in the cement matrix is nearly impossible, so that researchers aim to achieve ‘as close as possible’ dispersion state. In this study we used the most common method—sonication along with mechanical stirring, without any other dispersants, which probably led to the lower observed toxicity of nanoparticles in the selected models. Therefore, following the issues described above, nanoparticles were deliberately used in the sonicated suspension to correspond to their planned further application in cement based-composites. For that reason, no additional substances/stabilizers were applied for studies. Nanoparticles were tested in a way that they would be prepared prior the incorporation of cement-based building materials. 

Gained results show that nanomaterials did not show expected toxicity in studied environment, while according to other authors, such nanomaterials should show relatively high toxicity [11,35,36,37]. Our results showed that toxicity was decreased in a way so that minimal inhibitory concentration (MIC) or minimal bactericidal concentration (MBC) values could not be established. This could be associated with the dispersion of nanomaterials, their size, composition, and purity. As mentioned above, it is known that dispersion through sonication produces limited efficiency in dispersing the nanostructures; nevertheless, this method is widely used by researchers in this field [28,33,36]. 

These substances do not act similarly to antibiotics that can be transferred by diffusion. Nanoparticles are not single molecules, and therefore their diffusion is minimalized. In current studies, we used pure nanoparticles, so that solutions should not contain free ions that can be responsible for the higher toxicity of nanomaterials, which may explain the relatively low observed toxicity. This problem was described by Beer et al. [38], who indicated that ions can give false positive results regarding toxicity. Nanomaterials bought for this study were purchased and tested to decrease the possibility of such action. This paper revealed a probable cause of high efficiency of nanomaterials from the ‘green’ synthesis, which is, e.g., based on reduction from AgNO_3_ in the case of Ag nanoparticles. If nanomaterial is not purified after synthesis, its solution contains ions that additionally increase the toxicity. This could explain relatively low bactericidal efficiency gained in our studies, for the experiments were conducted on purified nanoparticles suspended in ultrapure water. On the other hand, such results show that pure and partially agglomerated nanomaterials may not be accessible to cells, and thus the observed toxicity would be lower.

Relatively weak toxicity gained in the most of conducted experiments could be attributed to the agglomeration of nanoparticles [30]. However, this process can occur in case of preparation of nanomaterials to the cement-based composites. As described in the introduction section, incorporation of nanomaterials to the prior incorporation of cement-based composites has certain conditions, including limited amount of water and lack of surfactants. Therefore, dispersion that was obtained in this work was maximum possible dispersion that can be achieved using this method [30,32,33,34]. We assume that the agglomerated material descended at the bottom and thus was less accessible to microorganisms, despite the fact that cultures were led with shaking. This observation can be supported by the results gained from biofilm formation assay. The biomass in samples with *E. coli* was significantly decreased by all nanomaterials. On the other hand, *P. aeruginosa* biofilm biomass was comparable to the control sample. The difference between these strains could be the location of formed biofilms. *E. coli* tends to produce it mostly at the bottom of plate, while *P. aeruginosa* directs most of the biomass into the surface. *S. aureus* was significantly affected only by ZnO nanoparticles. This bacterium also produces biofilm at the bottom of well; thus, this finding showed that nanoparticles in general could be inaccessible to cells [39,40,41,42]. 

As described in the introduction section, currently there are no standards for non-photocatalytic cement-based surfaces. Another problem regards the microbiological material that is used for studies, and which is often undiversified [3,5,8,17]. Furthermore, most authors do not discuss the specific traits of the strains that they used. The information is often limited only to the genus, designation of group, or simply GenBank number, referring only to one gene coding 16S rRNA that is being used to determine the genus [3,35]. In current microbiology, sequence coding 16S rRNA is still useful, although it may be not enough for the accurate determination of taxonomic position of strains [43,44]. From the microbiological point of view, two strains in the same genus can express different features. For example, *Escherichia coli* strains show different adaptational traits that include the variability in biofilm formation capacity and the ability to adhere to surface or possess genes responsible for antibiotic resistance. The genome of some strains may also contain bacteriophage coding toxins such as Stx toxin [45,46,47]. Moreover, it is not advisable to provide only the name (or acronym) of strain, because it may have many derivates such as *E. coli* K-12 [48]. The NCBI taxonomy browser provides over 3000 hits when one searches for *Escherichia coli* [49]. Such issues create difficulties in validation and analyzing the test results. Here, it was shown in only four *E. coli* strains that bacteria possessing different genetic profile could differently react to nanoparticles, which means that the evaluation of nanomaterials in terms of their antimicrobial activity should be supported by not only gathering the knowledge of the used strains, but it should also be executed on strictly selected or multiple strains from the same genus. This is also a good reason for organizing microbiologists into teams to evaluate antimicrobial activity such as in the article by Piszczek et al. [12], in which the authors used a strain of widely known reference. Therefore, particular endeavors should be directed in future towards developing methods and selecting strains that will be representative.

It should be highlighted that the gathered evidence does not undermine the known toxicity of metal oxide nanoparticles on microorganisms. The main aspect considers the necessity to design the standardized tests for evaluation of nanostructures that includes the planned application to the cementitious composite materials.

## 4. Materials and Methods

### 4.1. Materials

Nanoparticles were provided by Sigma Aldrich (MERCK, Darmstadt, Germany). Nanooxides selected for the experiments were Al_2_O_3_, CuO, Fe_3_O_4_, and ZnO. 

Reference strains—*Escherichia coli* ATCC^®^ 8739™, *Staphylococcus aureus* ATCC^®^ 25923™, *Staphylococcus aureus* ATCC^®^ 6538™ (for biofilm formation), *Pseudomonas aeruginosa* ATCC^®^ 27583™, and *Candida albicans* ATCC^®^ 10231™, were used for biological studies. Results from growth kinetics studies were compared with three other *E. coli* strains—*E. coli* MG1655, and two of its derivates—genetically modified *E. coli* MDS42 and *E. coli* MDS69, provided thanks to Dr. Ildikó Karcagi, from the Synthetic and Systems Biology Unit in the Institute of Biochemistry, in the Biological Research Centre of the Hungarian Academy of Sciences, Szeged, Hungary. All genetic modifications in these strains are listed in article by Karcagi et al. [50].

### 4.2. Physiochemical Evaluation of Nanomaterials

All nanomaterials were purchased from Sigma-Aldrich (MERCK, Darmstadt, Germany). For the preparation of experimental suspensions, sonicated nanomaterials were used without any further modification. Nanomaterials were prepared in the same manner as used in the microbiological studies (description below). The nanomaterials were investigated by transmission electron microscopy (Fei, Tecnai G2 F20 S Twin with energy dispersive X-ray spectroscopy, Thermo Fisher Scientific, Waltham, MA, USA). The crystalline structure and chemical composition of the samples was studied by X-ray diffraction. The XRD measurements were performed with a PRO X-ray diffractometer (X’Pert PRO Philips diffractometer, Co. Ka radiation, Almelo, Holland). The nanomaterials surface area was measured based on the N_2_ adsorption/desorption isotherms (Quantachrome Instruments, Quadrosorb SI, Boynton Beach, FL, USA). The specific surface area was calculated by the Brunauer-Emmett-Teller (BET) method. 

### 4.3. Preparation of Nanomaterials for Microbiological Studies

The suspensions of nanomaterials were prepared from powder in ultrapure (PCR grade) water, in stock concentration of 1000 µg/mL. Suspension was sonicated for 45 min along with high speed mechanical stirring. Basic working concentration of nanomaterials was 100 µg/mL, which was selected based on optimization experiments and literature. Lower concentrations gave marginal effects, while higher concentrations created problems with background noise in experiments. Nevertheless, chosen concentration was considered as toxic for microorganisms [11,35,36,37]. It should be highlighted that the further use of nanomaterials requires their addition to the cement-based composites. Therefore, concentration was kept on the level that has antimicrobial potential and at the same time could be used as admixture to cement-based composites. Every experiment was conducted with the same nanomaterial suspension in order to exclude the variability of preparation, which could affect the results.

#### 4.3.1. Growth Kinetics

Overnight cultures of studied strains were inoculated in ratio 1:200 to fresh Tryptone Soya Broth (TSB) liquid medium containing nanomaterials in concentration 100 µg/mL or ultrapure water in control sample. Cultures were led at 30 °C in orbital shaker incubator, with shaking at 140 rpm. Growth kinetics curves were obtained by measuring the optical density (600 nm) of liquid culture every 1 h for 10 h. 

#### 4.3.2. Toxicity Studies

24-h toxicity studies were performed in 96-well transparent polystyrene plates with a flat bottom. Overnight cultures were inoculated i ration 1:200 to fresh TSB medium containing 100 µg of studied nanoparticles per mL or ultrapure water in controls. Absorbance at wavelength of 600 nm was measured straight after preparation step and second time after 24 h. Due to the possible background noise caused by nanomaterials, each well was measured 4 times in four different spots. Furthermore, all necessary controls were applied including medium alone control, medium with nanomaterial, and medium with addition of water. 

4-h toxicity studies were conducted according to Ivask et al. [23]. Briefly, an overnight culture of bacteria and fungus were inoculated to fresh TSB medium and incubated at 30 °C until log phase was reached. In the next step, cells were centrifuged (10 min. at 3500 rpm) and resuspended in ultra-pure water. Nanomaterials were suspended in ultrapure water, sonicated for 30 min, and added to cells, reaching final concentration of 100 µg/mL. Afterwards, samples were kept at room temperature for 4 h without access to light. Post incubation, cells were diluted in serial dilutions method, and 100 µL was spread on TSA agar plates. Cultures were kept at 37 °C overnight. Colonies were counted after 18 h. The evaluation of colonies included comparison of morphology with control plates. In every case, inoculation was executed in three repetitions. Experiments were replicated in order to confirm gained tendencies of results. 

#### 4.3.3. Influence on Biofilm Formation

Biofilm formation studies were prepared in 96-well transparent polystyrene plates with round bottom. Each well was filled with 120 µL of fresh TSB medium and 15 µL of nanomaterial suspension (or ultrapure water in control samples). Suspensions of nanomaterials were prepared in a way that enabled them to reach final concentrations at 100 µg/mL. Afterwards, wells were inoculated with 15 µL of overnight culture of selected microorganisms. The plates were incubated for 24 h at 30 °C. Post incubation, plates were washed three times with PBS, and each well was filled with fresh TSB medium with the addition of 10% alamarBlue^®^. Afterwards, plate was incubated at 30 °C up to 4 h. Fluorescence were measured on BioTek Synergy HTX (BioTek Instruments, Winooski, VT, USA) (*λ*_ex_ = 520 nm; *λ*_em_ = 590 nm) in order to determine the viability of cells forming biofilms. In the next stage, plates were rinsed three times with deionized water, and biofilms were fixated with methanol for 15 min at room temperature. After that time, plates were emptied, air dried, and filled with filtered crystal violet (1% *w*/*v*). Staining lasted 15 min, while the plates were kept at room temperature. In the last stage, plates were rinsed with tap water and air dried. Biomass was decolorized with ethanol:acetone (8:2 *v*/*v*) solution, adding 200 µL for each well. Finally, 100 µL was thrice pipetted and transferred to a new 96-well flat-bottom plate. Absorbance at 570 nm was read on m200 PRO (Tecan, Männedorf, Switzerland) microplate reader. 

#### 4.3.4. Statistical Analysis

The results were statistically analyzed using one-way ANOVA with Tukey’s test as post-hoc testing. *p* < 0.05 were considered statistically significant. Assumptions for the ANOVA were checked for the each set of data. 

## 5. Conclusions

The evaluation of nanomaterials should consider their further application and characteristics. Poor dispersion can be the main technological problem in endeavors to use nanoparticles as antimicrobials. The evaluation of nanomaterials for cement-based construction materials should include various microbial strains, including different strains from the same species, for they can show a variable response to nanomaterials. Finally, based on in vitro studies, metal oxide nanoparticles may not be efficient at preventing microbial growth when unproperly dispersed, which will likely be the case in cement mortars and concretes. Therefore, the standard procedure including mixing and sonication (even though it is beneficial for improving other properties of cement-based composites, e.g., strength and durability) may not be satisfactory for providing certain antimicrobial properties. Thus, the development of new methods improving the dispersion of nanomaterials should be sought. Furthermore, the differences in gained results between studied bacteria and fungi make it necessary to develop a standardized approach for their testing in regard to planned application of nanomaterials.
